# Effectiveness of case management in the prevention of COPD re-admissions: a pilot study

**DOI:** 10.1186/s13104-017-2946-5

**Published:** 2017-11-25

**Authors:** Annelies E. van Eeden, Ingrid van de Poll, Gertrud van Vulpen, Tim Roldaan, Wies Wagenaar, Melinde R. S. Boland, Ron Wolterbeek, Niels H. Chavannes

**Affiliations:** 1 Department of Public Health and Primary Care, Leiden University Medical Centre, PO Box 9600, 2300 RC Leiden, Netherlands; 2 Lung Alliance Netherlands, Amersfoort, Netherlands; 3 Medical Coordinating Centre Flevoland, Lelystad, Netherlands; 4 MC Group, Lelystad, Netherlands; 5 Institute for Medical Technology Assessment, Erasmus University Rotterdam, Rotterdam, Netherlands; 6 Department of Medical Statistics and Bioinformatics, Leiden University Medical Centre, Leiden, Netherlands

**Keywords:** Case management, Chronic obstructive pulmonary disease, Hospitalization, Quality of life

## Abstract

**Background:**

Chronic obstructive pulmonary disease (COPD) exacerbations are associated with high disease burden and costs, especially in the case of hospitalizations. The overall number of hospital admissions due to exacerbations of COPD has increased. It is remarkable that re-admissions account for a substantial part of these hospitalizations. This pilot study investigates the use of case management to reduce re-admissions due to COPD.

**Methods:**

COPD patients with more than one hospitalization per year due to an exacerbation were included. The participants (n = 10) were closely monitored and intensively coached for 20 weeks after hospitalization. The case manager provided care in a person-focused manner. The case manager informed and supported the patient, took action when relapse threatened, coordinated and connected primary and secondary care. Data of 12 months before and after start of the intervention were compared. Primary outcome was the difference in number of hospitalizations. Secondary outcomes were health-related quality of life (measured by the Clinical COPD Questionnaire, CCQ) and dyspnoea (measured by the MRC Dyspnoea Scale).

**Results:**

The incidence rate of hospitalizations was found to be 2.25 times higher (95% confidence interval [CI] 1.3–3.9; *P* = 0.004) 12 months before compared with 12 months after the start of case management. COPD patients had a mean CCQ score of 3.3 (95% CI 2.8–3.8) before and 2.4 (95% CI 1.9–2.8) after 20 weeks of case management; a difference of 1.0 (95% CI 0.4–1.6; *P* = 0.001). The mean MRC scores showed no significant differences before (4.3; 95% CI 3.7–4.9) and after the case management period (3.9; 95% CI 3.2–4.6); a difference of 0.4 (95% CI − 0.1 to 0.9; *P* = 0.114).

**Conclusions:**

This pilot study shows that the number of COPD hospital re-admissions decreased significantly after the introduction of a case manager. Moreover, there was an improvement in patient-reported health-related quality of life.

**Electronic supplementary material:**

The online version of this article (10.1186/s13104-017-2946-5) contains supplementary material, which is available to authorized users.

## Introduction

Exacerbations of chronic obstructive pulmonary disease (COPD) are associated with high disease burden and costs, especially in the case of hospitalizations [1]. In the Netherlands, the number of hospital admissions due to COPD exacerbations has increased [2]. However, it is generally assumed that many hospitalizations are avoidable with good ambulatory care [3]. Nevertheless, this type of outpatient care requires a multidisciplinary approach and good interaction with the patient [4].

Lung Alliance Netherlands (LAN) is a federated association in the field of lung diseases and coordinates the National Action Programme on Chronic Lung Diseases (NACL). The NACL should ensure improvement of prevention and care for lung patients in the Netherlands. One of the main goals is to reduce the number of hospital admission days due to COPD by 25% [5].

Because re-admissions account for a substantial part of COPD hospitalizations, strategies are needed to avoid these re-admissions. Surprisingly, there is a paucity of evidence to guide clinicians on how to prevent these re-admissions [6, 7].

Prieto et al. systematically reviewed five randomized clinical trials assessing interventions to reduce re-admissions following COPD hospitalizations and concluded that there is inadequate information to recommend specific strategies to reduce the risk of hospitalizations [8].

Many factors are associated with COPD re-admissions. Besides disease specific factors, there are factors not specific to COPD, like comorbidities and psychosocial factors [9, 10]. It was hypothesized that a person-focused manner may be more efficient in preventing re-admissions. In a person-focused approach, the whole person is considered and not just the disease [8, 11]. Case management is considered as a promising intervention, since care could be individualized in this model [4].

Therefore, this study investigates the effects of case management on COPD re-admissions and whether this intervention improves health-related quality of life and dyspnea.

## Methods

### Setting

This pilot study within the NACL [5] was performed between 2012 and 2014 in the Dutch MC Zuiderzee hospital in Lelystad. Ethical approval was not required.

### Participants

Patients with COPD according to the GOLD criteria [1], with > 1 hospitalization in the last 12 months due to exacerbations, were included. The only exclusion criterion was severe psychiatric disorder(s) hindering COPD case management. All participants gave written informed consent.

### Intervention

During 20 weeks after hospitalization, each participant was intensively coached and monitored by a case manager. The case manager was a nurse specialized in COPD, who received training in person-focused care, coaching and strategies associated with self-management education.

The initial contact took place during hospitalization. Main goal of this contact was to assess respiratory medications and inhaler technique prior to discharge.

After discharge, the case manager visited the patients five times at home. Each visit lasted approximately 40 min. If the patient cancelled an appointment, the case manager contacted the patient to check whether the patient still wanted to participate and if so, they made another appointment. The case manager provided care in a person-focused manner. This approach considered the whole person, rather than focusing on the illness.

During the home visits, the patients were monitored regarding physical, mental, emotional and social well-being, living situation, social network, therapy adherence, coping, self-management and care needs. The Clinical COPD Questionnaire (CCQ) [12, 13] and the Medical Research Council Dyspnea Scale (MRC) [14] were administered. The case manager investigated all needs and made an individualized strategy for each patient. Proper communication was used to create conditions which promote self-management and a spirit of collaboration. The case manager inventoried exacerbating factors, prepared a written exacerbation action plan to recognize and manage exacerbations, took action when relapse threatened and supported the patient. The patient received information about (dealing with) COPD as a disease, medication, inhalation technique, physical activity, nutrition, and smoking cessation. Where appropriate, family members were involved in the education process as well. The case manager served as a contact and patients were invited to call the case manager if they had any concerns. Furthermore, the case manager scheduled multidisciplinary meetings and coordinated and connected primary and secondary care.

### Statistical analysis

An intention-to-treat analysis was performed. Data acquired before and after start of the intervention were compared. Primary outcome was the difference in number of hospitalizations during 12 months. Secondary outcomes were health-related quality of life as assessed with the CCQ [12, 13] and dyspnea as measured with the MRC [14]. The last CCQ and MRC measurements of patients that dropped out during the study period were carried forward in the corresponding analysis.

Generalized estimating equation analysis was used, thereby taking into account the correlation within a patient between the periods. For the primary outcome we applied repeated measures Poisson regression. For the secondary outcomes we used the normal distribution.

The SPSS version 20 was used for statistical analyses. We considered a P value of less than 0.05 to be significant.

### Cost analysis

A cost analysis was performed.

## Results

Of the 11 selected COPD patients, one patient was excluded due to an anxiety disorder and refusal to allow visits from the case manager. Table 1 presents baseline characteristics of the included patients. During the intervention period, one participant died due to complications of COPD and another patient dropped out because they no longer wished to participate. Two home-visits were cancelled because these patients were admitted to the hospital. The case manager subsequently visited these patients during their hospitalization.

**Table 1:** Baseline characteristics of the study population (n = 10)

Men, %	20
Age in years	62.9 (9.6)
Pulmonary function	
FER%	41.7 (15.3)
Predicted FEV1%	45.3 (28.5)
GOLD stage, %	
1 Mild: FEV1 > 80%	10
2 Moderate: 50% ≤ FEV1 < 80%	30
3 Severe: 30% ≤ FEV1 < 50%	10
4 Very severe: FEV1 < 30%	50
CCQ total score	3.3 (0.8)
MRC	4.3 (1.1)

Values are presented as means (standard deviations), unless stated otherwise

*FER* forced expiratory ratio (FEV1/FVC × 100%), *FVC* forced vital capacity, *FEV1* forced expiratory volume in 1 s, post-bronchodilator, predicted according to age and height; *GOLD* global initiative for chronic obstructive pulmonary disease *CCQ* Clinical COPD Questionnaire; *MRC* Medical Research Council Dyspnea Scale

The incidence rate of hospitalizations was 2.25 times higher 12 months before compared with 12 months after start of case management, as shown in Table 2. There was no difference in mean duration of hospitalizations. After the intervention, the number of days of hospitalization per patient/year decreased on average by 13.7 days.

**Table 2:** Differences in COPD hospitalizations, CCQ and MRC scores before and after the case management period

Outcome	Usual care	Case management	Mean difference	*P* value
Hospitalizations, rate per year	3.4 (2.6–4.5)	1.5 (0.9–2.5)	2.25 (1.3 to 3.9)^a^	0.004
Duration of hospitalization, days	7.49 (5.8–9.2)	7.53 (5.1–10.0)	0.04 (− 1.7 to 1.8)	0.961
Total days in hospital per patient per year	26 (17.2–34.8)	12.3 (5.3–19.4)^b^	− 13.7 (− 24.5 to − 2.8)	0.013
CCQ total score	3.3 (2.8–3.8)	2.4 (1.9–2.8)^c^	− 1.0 (− 1.6 to − 0.4)	0.001
CCQ: symptoms domain	3.3 (2.6–3.9)	2.5 (2.0–3.0)^c^	− 0.7 (− 1.3 to − 0.2)	0.009
CCQ: functional domain	4.1 (3.5–4.6)	2.8 (2.2–3.3)^c^	− 1.3 (− 2.1 to − 0.6)	0.001
CCQ: mental domain	2.0 (0.9–3.1)	1.2 (0.5–1.9)^c^	− 0.8 (− 1.8 to 0.2)	0.117
MRC	4.3 (3.7–4.9)	3.9 (3.2–4.6)^c^	− 0.4 (−0.9 to 0.1)	0.114

Values are presented as means (95% confidence interval), unless stated otherwise

*CCQ* Clinical COPD Questionnaire, *MRC* Medical Research Council Dyspnea Scale

^a^Rate ratio

^b^One patient included who died after 3 months; value multiplied by 4

^c^Mean value after 20 weeks of case management, includes last measurements of deceased patient (after 8 weeks) and of dropped-out patient (after 12 weeks)

Mean CCQ scores improved by 1.0 point after 20 weeks of case management. The improvement exceeds the clinically relevant improvement of 0.4 points [13]. Mean MRC scores showed no significant differences.

### Cost analysis

In this pilot study, the costs for the use of a case manager were 1835 euro (24 h; 76 euro/h) per patient/year. Taking implementation costs into account, the total costs of the case management program were approximately 3350 euro per patient/year.

In 2013 in the Netherlands, COPD hospitalizations cost 498 euro per day according to the ‘national guidelines for pharmacy-economic research [15]’ and after inflation adjustment [16]. With 6.7 fewer hospital days per patient/year, the intervention covers costs. The present study shows that, after case management, days in hospital decreased by 13.7 days. The declined hospital costs exceed the costs of case management, resulting in a net saving of €3473 per patient/year.

## Discussion

In the present study, the number of hospitalizations due to COPD exacerbations in patients with frequent re-admissions decreased by 56% after introduction of a case manager, which contributes to one of the main goals of the NACL [5]. Our results are in line with the idea that pro-active guidance is necessary for good ambulatory care [17]. The case manager supports the patient in several ways, which eventually improves the patient’s self-management. Our patients were positive about the intervention. They indicated that they felt more independent and had more control over their disease process after the case management period. Their knowledge about disease and medication improved. They had a more positive outlook on life, were less anxious and accepted their situation more. Patients learned to take action at the right time, such as contacting their general practitioner and using medication appropriately. Besides, patients experienced social progress. Some participants indicated that they had a better relationship with their partner and others enjoyed their grandchildren more.

The health-related quality of life improved with clinically important differences. Since feelings of anxiety and depression are common among COPD patients and social factors play a role in some hospitalizations [5, 9, 10], psychosocial factors need attention at an early stage of a case management program. A person-focused approach (instead of a patient-focused approach) as used in this study is recommended.

No differences were found in dyspnea, perhaps because case management does not directly affect lung function. After the intervention, patients experienced the same level of severity of dyspnea, but felt better able to deal with their disease as represented by an improvement on functional domain of the CCQ.

### Limitations

This pilot study with one case manager was designed as a proof of concept and included only 10 participants. The results need to be interpreted with caution. Even in our small study group the results are convincing. However, the effect sizes might be overestimated, as regression to the mean was not taken into account. Therefore, future studies should include a control group. Nevertheless, the included participants had been repeatedly admitted for several years and were expected to have a similar number of hospitalizations with unchanged care. The introduction of a case manager seems to break the vicious cycle of re-admissions and would definitely have an effect on the number of hospitalizations. Due to the comprehensive person-focused approach, it is difficult to pinpoint the particular components of the case management that led to the effect.

The substantial decrease in hospitalizations might be explained by the inclusion of COPD patients with > 1 hospitalization per year. Case management may also prove effective in other patient groups, but may be most (cost-) effective in those subgroups with considerable room for improvement.

COPD severity could play a role as well. Since patients with a higher burden of disease have more room for improvement, they might benefit more from case management. Due to the small sample size of our pilot study, we were unable to do subgroup analyses to analyze the impact of COPD severity. It would be interesting to investigate this in larger studies.

In Lelystad, a larger project has been started to confirm the present results. The number of hospitalizations increased after the case management period of 20 weeks and participants appreciated a second period with less frequent monitoring after the intensive monitoring period of 20 weeks. Based on these experiences, the case management period will be extended from 20 weeks to 1 year. Participants will be visited quarterly during the second half year of the intervention.

Besides, the multidisciplinary meetings will be changed in less frequent conference calls, since the meetings were encountered as time consuming by the involved care professionals.

Our cost-analysis showed that the case management program resulted in a net saving. It is expected that the total costs of case management will decrease in the coming years, especially due to less implementation costs. Consequently in the future, a break-even result may be reached with a smaller reduction in hospital days (< 6.7 days). A reduction in hospitalizations is favorable for patients and health insurance companies, however, hospitals may lose income and employees may lose their job. A transition could be introduced whereby hospital nurses are retrained as case managers. There might also be a role for health insurers to ‘reward’ care groups that achieve fewer hospitalizations due to effective case management. A funding model for this transmural care needs to be found (Additional file 1).

## Conclusions

In conclusion, the current data show that case management might be a valuable intervention to reduce COPD hospitalizations and to improve quality of life. More research is necessary to confirm these findings.

## Additional file

**Additional file 1.** Dataset manuscript effectiveness of case management in the prevention of COPD re-admissions.

## Abbreviations

CCQ: Clinical COPD Questionnaire
CI: confidence interval
COPD: chronic obstructive pulmonary disease
GOLD: global initiative for chronic obstructive lung disease
LAN: Lung Alliance Netherlands
MRC: Medical Research Council Dyspnea Scale
NACL: National Action Programme on Chronic Lung Diseases
